# Maintaining Excellent Mechanical Properties via Additive Manufacturing of Low-N 25Cr-Type Duplex Stainless Steel

**DOI:** 10.3390/ma16227125

**Published:** 2023-11-10

**Authors:** Jianguo He, Jiesheng Lv, Zhigang Song, Changjun Wang, Han Feng, Xiaohan Wu, Yuliang Zhu, Wenjie Zheng

**Affiliations:** Research Institute of Special Steels, Central Iron & Steel Research Institute Co., Ltd., Beijing 100081, China; hejianguo@nercast.com (J.H.); lvllvlv@foxmail.com (J.L.); wangchangjun@nercast.com (C.W.); fenghan@nercast.com (H.F.); wuxiaohan@nercast.com (X.W.); zhuyuliang@nercast.com (Y.Z.); zhengwenjie@nercast.com (W.Z.)

**Keywords:** selective laser melting, duplex stainless steels, nano-inclusion, microstructure, mechanical properties

## Abstract

Duplex stainless steel (DSS) exhibits good mechanical properties and corrosion resistance, and has attracted more and more attention within the fields of both science and technology. However, the increasing levels of N and of Cr, Mo, etc., as alloying elements in DSS increase production difficulty. In particular, the N element increases the risk of Cr_2_N precipitation, which can seriously deteriorate the thermal plasticity of DSS, while increasing its strength. For this reason, a low-N-content 25Cr-type DSS was designed in order to adapt additive manufacturing processes. With regard to the nano-inclusions of oxide precipitation and effective grain refinement, and considering the benefits of selective laser melting fabrication, a low-N 25Cr-type duplex stainless steel with a 0.09 wt.% N content achieved high mechanical properties, with a yield strength of 712 MPa and an elongation of 27.5%, while the V-notch impact toughness was 160 J/cm^2^. The microstructure evolution and the reasons behind the improvement in mechanical properties will be discussed in detail.

## 1. Introduction

Duplex stainless steel (DSS) is composed of ferrite and austenite phases that exhibit a higher strength than conventional austenitic stainless steel, while the higher Cr, Mo, and N concentrations in DSS contribute to an increased and outstanding corrosion resistance compared to that of conventional austenitic stainless steel [1,2,3,4,5,6,7]. DSS also exhibits a unique resistance to intergranular corrosion in certain environments, making it irreplaceable in the petroleum, chemical, and marine [8,9,10,11] industries, among others. Compared to the 300-series austenitic stainless steels, duplex stainless steel can achieve higher strength and PREN values at similar or lower costs.

Although duplex stainless steel has certain advantages over austenitic stainless steel in terms of performance, there are still challenges facing the complete replacement of austenitic stainless steel by duplex stainless steel on the market when it comes to widely used application. The American Society for Testing Materials (ASTM) only includes seven types of cast duplex stainless steels in its standards, while there are hundreds of types of forged duplex stainless steels [8]. The slow cooling process during the cooling process after solidification leads to the formation of harmful secondary precipitates, such as CrN, Cr_2_N, sigma (σ) phase, or chi(χ) phase, which significantly affect both the mechanical properties and the corrosion resistance [11,12,13]. This makes it challenging to maintain the performance of duplex stainless steel in complex structural applications using traditional casting processes, which is an important reason behind the limited scope of application for duplex stainless steel.

In recent years, the development of additive manufacturing technology has introduced a new metal-forming process with extremely fast cooling rates [14,15,16,17,18,19,20,21]. The one-time forming method reduces spending on molds and allows for the manufacturing of more complex structural components, while avoiding issues like σ phase precipitation and grain coarsening during casting cooling [19,22,23,24,25,26,27,28]. The characteristics of additive manufacturing effectively address the issues of casting formation and the diversification of product forms in duplex stainless steel.

Based on the aforementioned research background, this work focuses on 2507-type duplex stainless steel with a lower N content (≤0.1 wt.%) and utilizes the laser powder bed fusion (L-PBF) method to fabricating 25Cr-type DSS with a high strength and plasticity and excellent toughness. The evolution of its microstructure at different stages is analyzed, and precipitation-strengthening and grain-refinement advantages are achieved through control of the composition and printing methods. This method is expected to provide an alternative composition optimization strategy for additive manufacturing processes for the application of duplex stainless steel.

## 2. Materials and Methods

**Figure 1:**
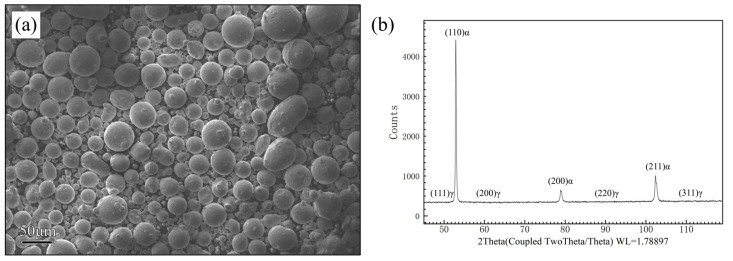
Powder morphology and phase information: (**a**) powder SEM image, (**b**) XRD diffraction pattern.

Argon-gas atomization was used to prepare 25Cr-type DSS powder with the size distribution of 15–50 μm. The main chemical composition of the powder was measured via inductively coupled plasma atomic emission spectrometry (ICP-AES), and the results are shown in Table 1. Figure 1a shows the morphology observed under scanning electron microscopy (SEM); the powder exhibits a smooth and round shape without satellite particles. The X-ray diffraction (XRD) analysis results for the powder are shown in Figure 1b, revealing a phase composition of 99% ferrite and 1% austenite, with no harmful phases detected.

The aforementioned powder was used for selective laser melting (SLM) on a DLM-280 metal 3D printer. The build process was performed on a 316 stainless steel substrate in a protective atmosphere of high-purity argon gas (99.9%). The specific sintering parameters were as follows: a laser input power (*P*) of 190 W, a laser spot diameter of 0.1 mm, a powder layer thickness (*h*) of 0.02 mm, line spacing (*t*) of 0.1 mm, a scanning speed (*v*) of 850 mm/s, and a bidirectional scanning pattern with a 45° angle between each layer. The calculated energy density, using Formula (1), was 117.65 J/cm^3^.

(1) $$E=\frac{P}{v\cdot h\cdot t}\;\;\;\;\;$$

**Figure 2:**
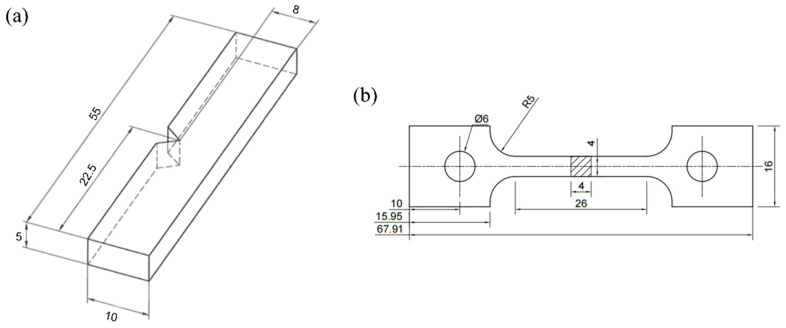
The dimensions of the specimens for testing mechanical properties: (**a**) the impact specimen dimensions; (**b**) the tensile specimen dimensions (unit: mm).

The built specimens were subjected to solid solution treatment at temperatures of 1100, 1150, and 1200 °C for 1 h each. Cube samples measuring 10 × 10 × 5 mm were used to characterize the microstructure, while rough impact samples with dimensions of 10 × 60 × 6 mm and rough tensile samples with dimensions of 20 × 70 × 6 mm were used to test the mechanical properties. The final dimensions of the impact and tensile specimens are shown in Figure 2a,b, respectively. The relative density of the specimens, using the Archimedes drainage method, was 99.87%.

Tensile testing at room temperature was performed using a WE300B tensile testing machine (supplied by Marxtest Technology Co., Ltd., Jinan, China) with a strain rate of 1 × 10^−3^ s^−1^. Impact testing at room temperature was conducted using a NI750 metal pendulum impact testing machine (supplied by NCS Testing Technology Co., Ltd., Beijing, China). After mechanical grinding and polishing, the square samples were immersed in a potassium permanganate solution at 50 °C for 3 h, and their microstructure was observed using a LEICA MEF4M optical microscope (supplied by Leica Microsystems Shanghai Ltd., Shanghai, China). The backscattered electron diffraction (EBSD) samples were immersed in a 10% alcoholic hydrochloric acid solution and subjected to electrolytic polishing at a voltage of 25 V for 30 s. EBSD characterization was carried out using a FEI Quanta650 field-emission scanning electron microscope (supplied by FEI Co., Ltd., Hillsboro, OR, USA), and the data were processed using Channel 5 software. Transmission electron microscope (TEM) samples were mechanically thinned to a thickness of 40 μm using silicon carbide paper and then further thinned using a dual-jet electropolisher at a voltage of −28 V and a temperature of −20 °C. The electrolyte consisted of 10% hydrochloric acid and 90% anhydrous ethanol. Observation was performed using a FEI TECNAI G2 F20 transmission electron microscope (supplied by FEI Co., Ltd., Hillsboro, American) operating at an accelerating voltage of 200 kV. X-ray diffraction experiments on the samples were conducted using a Bruker D8 Advance X-ray diffractometer (supplied by Bruker Co., Ltd., Billerica, MA, USA) with a tube voltage of 35 kV, a tube current of 40 mA, an incident wavelength of λ = 0.179 nm, and a scanning speed of 2°/min. The nitrogen content in the printed specimens was measured using an NCS ONH-5500 analyzer (supplied by NCS Testing Technology Co., Ltd., Beijing, China).

## 3. Results and Discussion

### 3.1. Microstructure

**Figure 3:**
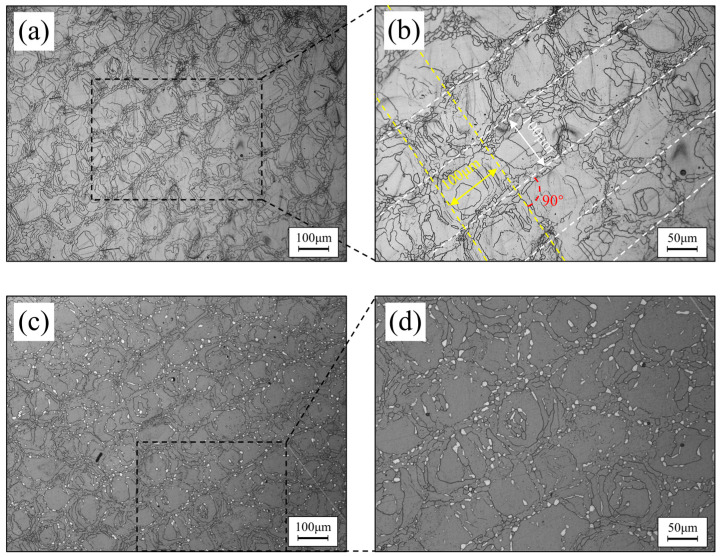
An optical microscope (OM) image of the specimen before and after heat treatment: (**a**) the specimen before heat treatment at 100× magnification, (**b**) the magnified microstructure of the selected area from (**a**), (**c**) the specimen heat-treated at 1200 °C for 1 h at 100× magnification, (**d**) the magnified microstructure of the selected area from (**c**).

Figure 3a shows the microstructure of the built cubic specimen without heat treatment, which reveals a mosaic-like regular structure. The magnified metallographic image in Figure 3b further reveals that the mosaic-like grid structure is arranged in a regular pattern, with 100 μm as the minimum structural unit. The core of the mosaic unit consists of larger grains, while the edges consist of smaller grains similar to recrystallized grains. The track width formed by the units is 100 μm, arranged in two directions at a 90° angle to each other. The aforementioned mosaic structure is mainly due to the special scanning method, where the laser spot radius is 100 μm, and the scan line spacing is also 100 μm. Due to the higher energy at the core of the laser, the powder in the core receives more energy compared to the powder at the edges, resulting in a difference in grain size between the core and the edges. At this point, the grain size distribution presents larger grains in the core and smaller grains on the sides. As the scanning progresses, after the completion of one layer, the scanning path of the next layer of powder is perpendicular to the previous layer at 90°. The laser energy input of the next layer, to a certain extent, affects the already-formed matrix in the previous layer, causing secondary heating and recrystallization, ultimately leading to the grid-like structure. The microstructure metallography of the specimen when heat-treated at 1200 °C for 1 h is shown in Figure 3c, and it is worth noting that the grid-like mosaic structure in the microstructure remains intact and regular after heat treatment, which is significantly different from the destruction of the grid-like structure that has been observed in previous studies after heat treatment [29,30,31,32,33]. The magnified microstructure shown in Figure 3d reveals that a certain amount of new phase has precipitated on the grain boundaries of the matrix phase and, even after 1 h of heat treatment at 1200 °C, the size remains small. The above microstructural characteristics are mainly attributed to the unique low-nitrogen (N)-composition design.

**Figure 4:**
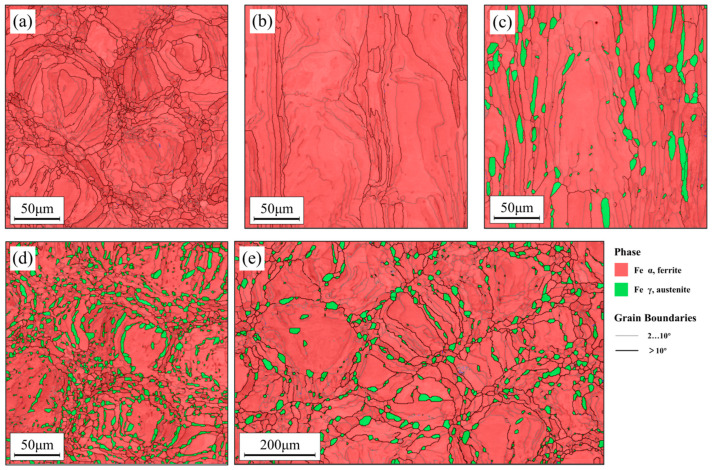
EBSD phase distribution maps: the front surface (**a**) and side surface (**b**) of the specimen before heat treatment, the front surface (**e**) and side surface (**c**) of the specimen heat-treated at 1200 °C for 1 h, the scan of the front surface (**d**) of the specimen heat-treated at 1100 °C for 1 h.

To further characterize the microstructural features before and after heat treatment, the phase distribution before and after heat treatment was statistically analyzed using EBSD. Figure 4a shows the EBSD phase map of the laser-irradiated surface before heat treatment, which predominantly consists of the ferrite phase. This is mainly due to the high temperature during the scanning process and the extremely fast cooling rate, which does not allow for the precipitation of austenite during the forming and cooling process. Figure 4b shows the EBSD phase map of the non-heat-treated scanning side, and it can be observed that each layer of ferrite has been melted by the laser into a solid structure with a width of approximately 100 μm. This solid structure will improve the impact toughness, which will be discussed in detail later. Figure 4c,e, respectively, show the phase distribution maps of the scanned front and side after 1 h of heat treatment at 1200 °C. A large amount of austenite precipitates at the grain boundaries of the ferrite, and some austenite precipitates at the low-angle grain boundaries within the ferrite. It can be observed that the size of the austenite precipitated on the high-angle grain boundaries is significantly larger than that precipitated on the low-angle grain boundaries within the grain. This is mainly because the high-angle grain boundaries have a higher energy, providing convenience for the rapid nucleation and growth of austenite. Figure 4d shows the phase distribution map of the scanned front after heat treatment at a lower solution temperature of 1100 °C for 1 h. It can be found that, compared to at 1200 °C, the amount of austenite increases significantly, and the austenite is distributed in a strip-like pattern connected to the grain boundaries of the ferrite. At a lower solution temperature, the morphological characteristics of the austenite phase have changed significantly compared to the discontinuous island-like distribution at a higher solution temperature.

### 3.2. Mechanical Properties

#### 3.2.1. Tensile Properties

**Figure 5:**
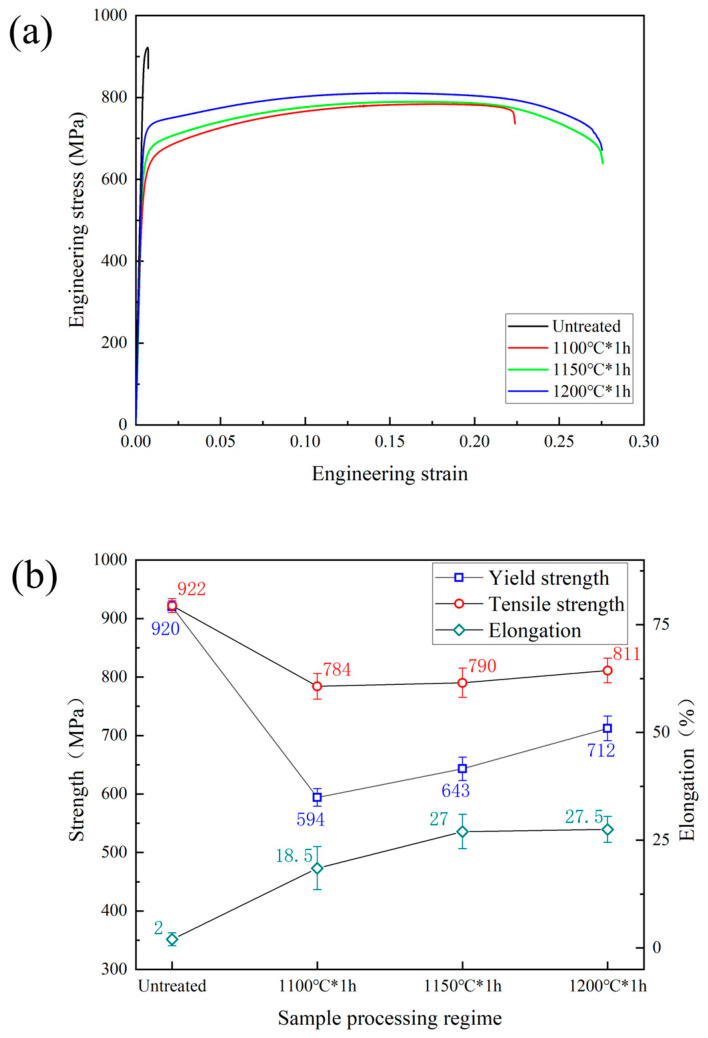
The tensile performance of the specimen: (**a**) the stress–strain curve, (**b**) the yield strength, tensile strength, and elongation at different treatment states.

Figure 5 shows the tensile properties of the sample before and after heat treatment. It can be observed that the sample has a very high yield strength (920 MPa) and tensile strength (922 MPa) in the state before heat treatment, but the elongation is only 2%. Concerning the tensile properties of the sample after heat treatment, it can be observed that, as the solution temperature increases, the strength and plasticity of the sample simultaneously increase. After solution treatment at 1200 °C, even with only a weak solid solution strengthening due to a N content of only 0.098%, the yield strength is still as high as 712 MPa. In previous studies, the yield strength of standard-grade (higher-N-content, compared to this paper) 2205 and 2507 duplex stainless steel, whether through casting or additive manufacturing, typically ranged from 600 to 660 MPa [15,34,35,36]. The test results obtained in this paper showed that the yield strength of the samples after a 1 h solution treatment at 1200 °C was significantly higher than that shown in the experimental results of previous studies.

#### 3.2.2. Impact Properties

**Figure 6:**
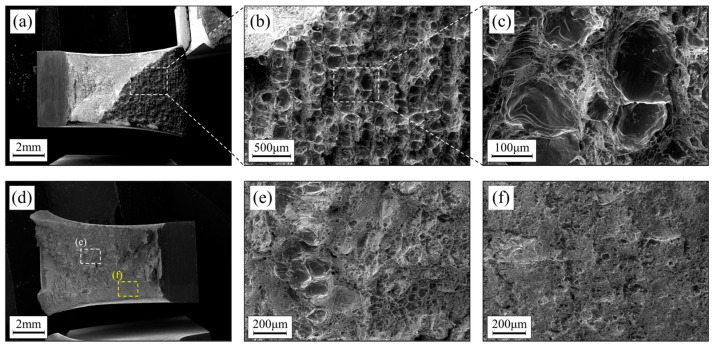
SEM images of the impact fracture surfaces: the fracture morphology of the specimen before heat treatment (**a**) and selected-area enlargement (**b**,**c**), the fracture morphology of the specimen heat-treated at 1200 ° for 1 h (**d**), and selected-area enlargement (**e**,**f**).

Table 2 presents the impact toughness values of the samples before and after heat treatment, compared with the impact performance of the 2707 BPF samples with similar processes. It can be observed that the impact toughness of the experimental steel in this study is significantly higher than that of the reference, and that the impact toughness value of the untreated samples is significantly higher than that in the results for the reference. Although the samples without heat treatment only show a 2% elongation during the tensile process, they exhibit high toughness values. SEM images of the fractured surfaces of the untreated samples at different scales are shown in Figure 6a–c. The unique fracture morphology is an important reason behind the higher toughness value of the samples. From the macroscopic fracture morphology in Figure 6a, it can be observed that the fracture surface of the sample is divided into two sides. The side near the V-notch (the whiter side) shows irregular cleavage fracture, while the other side exhibits a mosaic-like structure similar to its microstructure. The magnified images in Figure 6b,c reveal more clearly the complex fracture forms. In each small structural unit, the central large grains exhibit smooth cleavage fracture planes. The small grain area at the edge of the structural unit consists of a certain number of smaller dimples. The smaller grain size and multiple interfaces in this region partly hinder the propagation of cracks during the fracturing process, thus improving the impact resistance. On the other hand, as mentioned earlier, the microstructure on the sample’s side consists of ferrite arranged vertically as a whole. Cracks can only propagate through transgranular fracture within these ferrite regions, resulting in a fracture surface that is similar in appearance to the front surface’s grid-like structure. The coexistence of mixed microstructures with different grain sizes, and the integrity of the ferrite in the side structure, contribute to the complex fracture form and the high toughness value of the samples.

The SEM images of the fractured surface of the heat-treated impact samples are shown in Figure 6d–f. The macroscopic image in Figure 6d reveals a composite structure with smooth edges and an uneven core. The magnified image in Figure 6e of the core region shows a combination of dimples and cleavage planes, with some areas still exhibiting grid-like structures. The magnified image of the fracture edge in Figure 6f reveals cleavage planes. The structural differences between different regions of the fracture surface indicate a relatively poor continuity of crack propagation during the fracturing process, ultimately resulting in a more objective measure of the impact toughness.

## 4. Discussion

**Figure 7:**
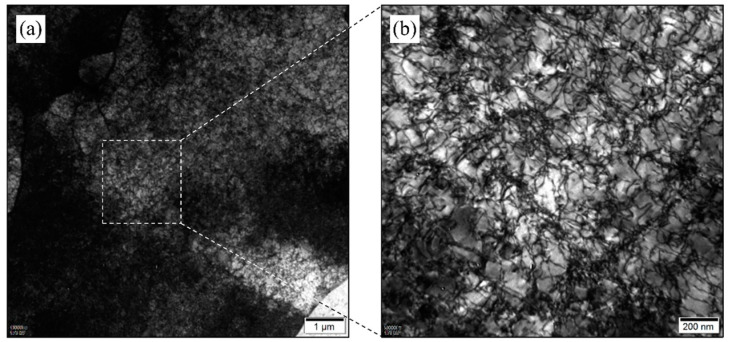
TEM image of the specimen before heat treatment (**a**) and selected-area enlargement (**b**).

The extreme tensile behavior of the untreated sample is mainly due to the presence of a ferrite structure in the untreated state. Typically, at room temperature, the nitrogen saturation solubility in ferrite is 0.007%, while most of the 0.098% nitrogen in the composition is dissolved in the ferrite, leading to a distorted ferrite lattice due to nitrogen oversaturation. Additionally, the repeated heating and rapid cooling during the printing process result in significant residual thermal stresses. The combination of these factors leads to a low plasticity and high strength in the untreated sample. The TEM characterization of the untreated sample confirms the above inference. Figure 7a shows the internal structure of the ferrite at the TEM scale. It can be observed that the interior of the undeformed ferrite is highly disordered. The magnified view in Figure 7b clearly shows a large number of randomly distributed and tangled dislocations within the ferrite grain. Even without deformation, the ferrite in this state is under a significant internal stress and, thus, fractures with only a small amount of deformation (2%).

As mentioned earlier, the yield strength of the samples after a 1 h heat treatment at 1200 °C reached values as high as 712 MPa, while still maintaining a 27.5% elongation. This anomalous mechanical performance is mainly due to the combined effect of the unique phase morphology of austenite under special composition and the precipitation of high-temperature oxide phases. At lower solution temperatures, the austenite content is higher, and a large amount of austenite forms continuous bands along the ferrite grain boundaries [37,38]. The softer and higher-content austenite leads to a decrease in yield strength, and the large distribution on the grain boundaries easily causes a deformation mismatch between the two phases, leading to fracture. Conversely, at higher solution temperatures, the austenite content is lower, and it is discretely distributed in island-like shapes along the ferrite grain boundaries. For this reason, there is less austenite with a smaller size and discrete distribution, which acts as a second-phase reinforcement by pinning on the ferrite grain boundaries, achieving an enhanced strength and plasticity.

**Figure 8:**
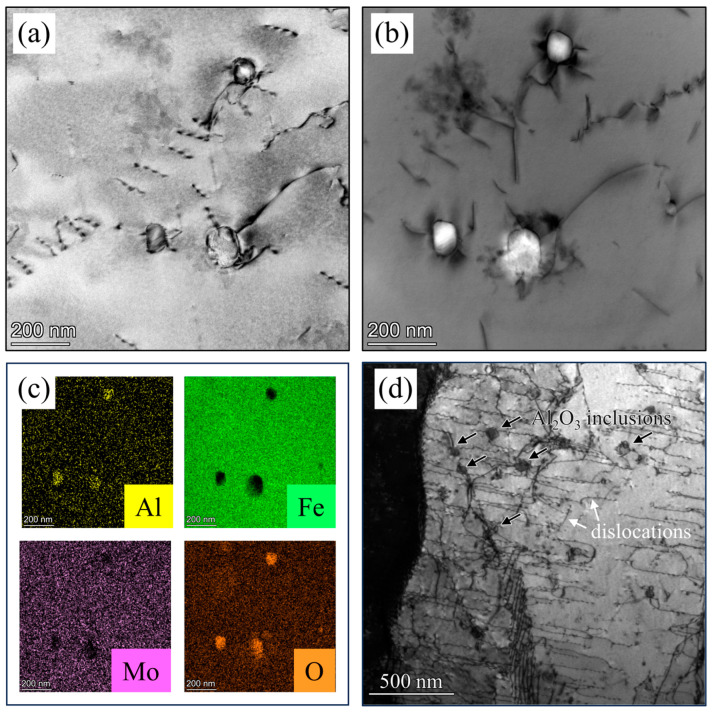
TEM micrographs of oxide inclusions in the specimen after 1 h heat treatment at 1200 °C: (**a**) the undeformed state, (**b**) the dark-field image of (**a**,**c**) the EDS mapping of (**a**,**d**) the deformed state.

In addition, in previous studies, the introduction of high-temperature oxide Al_2_O_3_ into the system also leads to an improvement in strength [33,39]. In traditional casting processes, the high-temperature-formed Al_2_O_3_ easily grows and producies inclusions during slow cooling, making it difficult to eliminate. However, in additive manufacturing processes, the extremely fast cooling rate prevents these high-temperature-formed Al_2_O_3_ from growing, and they ultimately become precipitates that are beneficial to mechanical properties. In this study, TEM observation also revealed fine dispersed circular Al_2_O_3_ precipitates, as shown in Figure 8a. The dark-field TEM image in Figure 8b provides a clearer view of the widely varying distribution sizes of Al_2_O_3_. To better illustrate the types of precipitates, Figure 8c shows the EDS mapping results of the region. It is evident that there is a clustering of Al and O elements in the precipitates. This type of precipitate strengthening is also one of the reasons behind the higher yield strength of the sample. We also characterized the uniform deformation zone of the samples after tensile fracture, using TEM. Figure 8d shows a TEM image of the deformed state, where a large number of dislocations are pinned by small Al_2_O_3_ inclusions in the ferrite. The interaction between the fine dispersoids of Al_2_O_3_ particles and dislocations hinders the initial movement of the ferrite during deformation, which contributes to the higher yield strength, compared to that under normal conditions.

In summary, through a unique composition design that breaks away from the conventional 0.24–0.32% nitrogen (N) content in 2507, a unique austenite phase morphology has been induced, while a regular lattice structure is maintained. With the reinforcement of oxide precipitates, the sample exhibits a high yield strength of 712 MPa, while still maintaining a good elongation of 27.5%.

## 5. Conclusions

In this study, a low-N 25Cr-type duplex stainless steel for additive manufacturing processes was fabricated through the L-PBF method. The product exhibits an excellent strength and ductility. The main conclusions are as follows:

1. When the strategy of alternating the layer scanning direction of 45° is used, the microstructure of the specimens shows a regular mosaic structure. The microstructure before heat treatment consists of a single ferrite phase. After heat treatment at 1200 °C, discrete and fine austenite precipitates at the ferrite grain boundaries, and the regular mosaic structure remains.

2. The unique morphology of the austenite phase, caused by the special low-N-composition design, along with the strengthening effect of oxide precipitation, leads to a high yield strength of 712 MPa after heat treatment in the experimental steel in this study, while a good elongation of 27.5% is maintained.

3. The specimen of additive-manufacturing low-N 25Cr-type DSS demonstrates an excellent V-notch impact performance, with a toughness value of 98.75 J/cm^2^ before heat treatment and approximately 160 J/cm^2^ after heat treatment.

## Figures and Tables

**Table 1:** The chemical composition of the 25Cr-type DSS powder ^1^.

Cr	Ni	Mo	N	Mn	O	Si	C	P	S
24.70	6.52	3.74	0.098	0.55	0.028	0.35	0.0050	0.0058	0.0029

^1^ S, C, and O elements were determined via the infrared absorption method.

**Table 2:** V-notch impact toughness.

Impact Toughness Value (aKV), J/cm^2^				
	Untreated	1100 °C × 1 h	1150 °C × 1 h	1200 °C × 1 h
This paper	98.75	158.75	161.25	160
Previous studies [26]	18	-	132	-

## Data Availability

The data presented in this study are available upon request from the corresponding author.
